# The Role of Estrogen Receptors in Health and Disease

**DOI:** 10.3390/ijms241411354

**Published:** 2023-07-12

**Authors:** Farzad Pakdel

**Affiliations:** Irset (Institut de Recherche en Santé, Environnement et Travail)—UMR_S 1085, EHESP, Inserm, Université de Rennes, F-35000 Rennes, France; farzad.pakdel@univ-rennes1.fr

Many biological and physiological events, including growth, development, and metabolism of reproductive and non-reproductive tissues in men and women, are regulated by estrogens and estrogen receptors (ERs). Over the past three decades following the discovery of the nuclear ERα, ERβ, and the membrane receptor, G protein-coupled ER (GPER also known as GPR30), multiple mechanisms of cellular action have been identified. These mechanisms involve either genomic actions through direct interaction with DNA, or non-genomic actions through the modulation of intracellular kinase cascades. ERs are closely linked to several serious human pathologies, such as breast, endometrial, ovarian, and prostate cancers, as well as osteoporosis, and coronary and neurodegenerative diseases. The Special Issue, “The Role of Estrogen Receptors in Health and Disease”, addresses some aspects of the potential role of ERs in various pathophysiological conditions by describing interesting results from in silico, in vitro, and in vivo experiments. Using Chromatin immunoprecipitation followed by DNA sequencing (ChIP-Seq), Collins et al. [1] have identified both ligand-dependent and ligand-independent binding sites of ERα throughout the genome. This study shows distinct ERα chromatin-binding profiles, depending on whether ERα is bound to the ligand or not, and has identified sites near many genes related to drug metabolism and drug therapy in the liver.

More than 70% of breast carcinomas express ERα, which drives the growth and progression of breast cancer cells, usually in the presence of estrogen. Typically, endocrine therapy using selective ER modulators is used clinically to inhibit the growth of these tumors. However, in 30–50% of cases, resistance to endocrine therapy occurs, with tumors acquiring the ability to proliferate in the absence of estrogenic stimulation. Clusan et al. [2] provide an overview of ERα mutations in breast cancer and their involvement in antiestrogen response and endocrine resistance. They also describe the influence of ERα synonymous mutations on the receptor folding and function in breast carcinogenesis. A study by Qi et al. [3] shows that berberine, a bioactive isoquinoline alkaloid, has an inhibitory effect on triple-negative breast tumors such as MDA-MB-231. They prove that berberine is a modulator of GPER that can inhibit cell viability, migration, and autophagy of MDA-MB-231 cells. Another aspect of the non-genomic action of ERs is reported by Lombardi et al. [4]. They demonstrate that activation of nuclear ERα and ERβ increases the phosphorylation of the protein kinase, SRC, in PC-3 prostate cancer cells. This non-genomic action of ERs triggering SRC- and PI3K/AKT-signaling pathways increases the tumorigenic potential of PC-3 cells by enhancing cell proliferation, migration, and tumor formation.

Chauvin et al. [5] review the importance of estrogen signaling in follicle development and its possible deregulation in human ovarian pathologies. The action of estrogens together with gonadotropins is essential for the quality of follicles, which depends on the correct growth and maturation of granulosa cells. ERβ is the major ER in these cells and several splice isoforms have also been found. The ratio of ER expression, particularly that of ERβ isoforms, could be crucial to granulosa cell proliferation and apoptosis and may be essential in the development of normal or tumor-derived follicles. On the other hand, cortical bone formation is regulated by Erα, which acts on progenitor cells. ERα knockout (ERKO) in female mice results in an impaired osteogenic response to exercise. However, Dirkes et al. [6] demonstrate that despite global loss of ERα, male ERKO mice are able to partially build bone in response to exercise. This would indicate that ERα is not required for the osteogenic response in male mice, unlike female mice. Thus, further studies are needed to explore additional cellular mechanisms that enable the osteogenic response to exercise in males. A study from Chuang et al. [7] sheds new light on the molecular mechanisms of estrogen-mediated skeletal development. This study reports that GPER is expressed in bone and likely plays an important role in regulating skeletal growth. The authors show that GPER mediates proliferation of bone mesenchymal stem cells through the cAMP/PKA/p-CREB pathway and then upregulates cell cycle regulators such as cyclin D1/cyclin-dependent kinase (CDK) 6 and cyclin E1/CDK2 complex.

The reduction of estrogens at menopause or alterations in the expression or activity of ERs in arteries, could contribute to the inability of estrogens to protect blood vessels during aging. Previous studies from Henrion and collaborators [8] have shown that membrane-anchored ERα participates in flow-mediated dilation (FMD), and that endothelial ERα contributes to improving FMD in young arteries. Using mice lacking ERα (Esr1^−/−^ mice) and C451A-ERα (lacking membrane ERα), Favre et al. [8] demonstrated that oxidative stress is a critical factor in the decline of FMD and an early ERα membrane defect implicated in vascular aging. In addition, Da Silva et al. [9] has contributed new information concerning the molecular, preclinical, and clinical implications of the cardiovascular effects of estrogen and the modulation of ERs as a potential therapeutic target for the treatment of cardiac dysfunction induced by myocardial infarction. Indeed, the activation of both nuclear ERs and GPER in the cardiovascular system has beneficial effects. This may be due to the stimulation of antioxidant enzymes, the reduction of proinflammatory proteins, and the enhancement of endothelial nitric oxide synthase and nitric oxide production. These mechanisms should be explored for the treatment of myocardial infarction and the prevention of subsequent heart failure.

In addition to their effects on reproductive behaviors, estrogens are now known to play an important role in cognition and mood through their interactions with multiple neurotransmitter systems, including dopamine and serotonin in the brain. Hwang et al. [10] provide an overview of this topic. They have summarized the emerging literature on ERs and psychiatric disorders in cellular, preclinical, and clinical studies. Current treatment strategies involving estrogens and estrogen signaling are also analyzed in the treatment of psychiatric disorders. Hirtz et al. [11] report a possible role for ERs in astrocytomas, which are the second-most-common glioblastoma. Epidemiological studies indicate that the incidence of gliomas is about 40% higher in men than in women, raising the question of the potential role of hormones in the occurrence of these tumors. The authors review recent data supporting the involvement of sex steroids in astrocytoma, which may be a hormone-sensitive tumor, and focus on the potential neuroprotective role of estrogens in this pathology.

Finally, the applications of different molecular modeling methods for the investigation of estrogens and xenoestrogens are summarized by Mazurek et al. [12]. As endocrine-disrupting chemicals (EDCs), xenoestrogens are environmental contaminants that perturb the endocrine function by interfering with any aspect of hormonal action. The authors review the interactions of estrogens and xenoestrogens with various proteins (protein-ligand docking), particularly enzymes involved in estrogen metabolism and ERs. The penetration of estrogens through lipid bilayers or their ability to adsorb to different materials is also examined by means of theoretical calculations. Avellaneda et al. [13] studied the mechanisms underlying the effects of EDCs such as HPTE and DES on embryonic thymocyte death and differentiation. Their data suggest that the adverse effects on embryonic thymocytes induced by HPTE and DES are not only dependent on ERα or GPER, but probably both. These results also provide evidence for a possible cooperation between ERs and T cell receptor signaling in mediating the adverse effects on embryonic thymocytes.

Once again, this Special Issue covers various aspects of the potential role of ERs. The thirteen studies reported here demonstrate only a few facets of the functions of ERs and their ligands in health and disease. Many thanks to all the authors and referees for their efforts in supporting this Special Issue.
